# A new dawn for monoclonal antibodies against antimicrobial resistant bacteria

**DOI:** 10.3389/fmicb.2022.1080059

**Published:** 2022-12-14

**Authors:** Marco Troisi, Eleonora Marini, Valentina Abbiento, Samuele Stazzoni, Emanuele Andreano, Rino Rappuoli

**Affiliations:** ^1^Monoclonal Antibody Discovery (MAD) Laboratory, Fondazione Toscana Life Sciences, Siena, Italy; ^2^Department of Biotechnology, Chemistry and Pharmacy, University of Siena, Siena, Italy; ^3^Fondazione Biotecnopolo di Siena, Siena, Italy

**Keywords:** monoclonal antibodies, bacteria, antimicrobial resistance, therapeutics, new technologies

## Abstract

Antimicrobial resistance (AMR) is a quickly advancing threat for human health worldwide and almost 5 million deaths are already attributable to this phenomenon every year. Since antibiotics are failing to treat AMR-bacteria, new tools are needed, and human monoclonal antibodies (mAbs) can fill this role. In almost 50 years since the introduction of the first technology that led to mAb discovery, enormous leaps forward have been made to identify and develop extremely potent human mAbs. While their usefulness has been extensively proved against viral pathogens, human mAbs have yet to find their space in treating and preventing infections from AMR-bacteria and fully conquer the field of infectious diseases. The novel and most innovative technologies herein reviewed can support this goal and add powerful tools in the arsenal of weapons against AMR.

## Introduction

The phenomenon called antimicrobial resistance (AMR), emerged as one of the leading public health threats of the 21st century. The review on antimicrobial resistance, commissioned in 2014 by the UK Government, estimated that AMR could kill 10 million people per year by 2050 (de Kraker et al., 2016; O'Neill, 2016). Despite this initial prediction, new statistical models estimated already 4.95 million deaths associated with bacterial AMR in 2019, highlighting a faster pace in the spreading of AMR pathogens worldwide (Murray et al., 2022). With antibiotics becoming increasingly obsolete, particular attention is turning to therapeutic monoclonal antibodies (mAbs) as an alternative approach to treat infectious diseases and address the global threat of AMR (Bebbington and Yarranton, 2008; Roca et al., 2015). Indeed, therapeutic mAbs have proven to be successful in treating several human viral pathogens including the respiratory syncytial virus (RSV), Ebola, Zika, Chikungunya, Influenza and more recently SARS-CoV-2 (Robinson et al., 2016; Sizikova et al., 2018; Kupferschmidt, 2019). Conversely, mAb therapy for bacterial pathogens are still struggling to find space in the battle against infectious diseases. On one side, mAbs could offer more effective ways of addressing antibiotic resistance and bacterial infections by targeting molecules specific only for pathogenic bacteria safeguarding the microbiota, activating the body's immune system resulting into a broader and more effective response, and reducing the toxicity associated with high antibiotic doses (Baker et al., 2018; Zurawski and McLendon, 2020). These benefits will ultimately result into countless lives saved and a drastic cut of hospital associated costs by reducing time of hospitalization from nosocomial infection. On the other side, many obstacles remain in the field of anti-bacterial mAbs. In contrast with mAbs against viral pathogens, Fc effector functions result to be essential for killing and bacterial clearance. In addition, bacteria expose hundreds of antigens on their surface, making the hunt for an optimal target a complex task. Moreover, in some cases, bacteria can form hardly penetrable biofilms and reside in body parts where mAbs are less likely to be distributed, thus, reducing their efficacy. These obstacles have limited the development of anti-bacterial mAbs resulting in only a few antibodies being tested in clinical trials (Table 1). While the technological leap of the last four decades have allowed to fine-tune mAb discovery and development, emerging approaches could allow to overcome the barriers which have so far limited the clinical application of mAbs against bacterial pathogens. This review examines the major past and present technologies used to discover and develop mAbs against bacteria. The principal focus is dedicated to technologies enabling effective mAbs against bacterial infections which could be used in the near future to support the fight against AMR.

**Table 1:** The table reports a list of mAbs against bacteria that have entered clinical trials.

**Bacterial species**	**Drug**	**Type**	**Target**	**Phase**	**Clinical trial ID**
Bacillus anthracis	Obiltoxaximab	Humanized IgG1	Protective antigen (toxin)	IV	NCT03088111
	Thravixa	Human IgG1		I	NCT01202695
	Valortim	Human IgG1		I	NCT00964561
	Raxibacumab	Human IgG1		IV	NCT02016963
Clostridium botulinum	XOMA 3ab	Mix of 3 humanized IgG1	Botulinum neurotoxin type B (toxin)	I	NCT01357213
	NTM-1632	Mix of 3 humanized IgG1		I	NCT02779140
Clostridium difficile	Actoxumab	Human IgG1	*C. difficile* toxin A (toxin)	III	NCT01241552
	Bezlotoxumab	Human IgG1	*C. difficile* toxin B (toxin)	III	NCT05304715
Escherichia coli	Edobacumab	Mouse IgM	LPS lipid A	III	/
	Nebacumab	Human IgM		III	/
	MAB-T88	Human IgM		III	/
STEC^*^	Shiga toxin MAbs, cαStx1 & 2	Mix of 2 humanized IgG1	*E. coli* Stx1 & Stx2	II	NCT01252199
Pseudomonas aeruginosa	Aerucin	Human IgG1	*P. aeruginosa* alginate	II	NCT02486770
	Panobacumab	Human IgM	*P. aeruginosa* LPS O11	II	NCT00851435
	KB001	Human PEGylated Fab	*P. aeruginosa* PcrV	II	NCT01695343
	MEDI3902	Bispecific human IgG1	*P. aeruginosa* PcrV and Psl	II	NCT02255760
Staphylococcus aureus	Salvecin, AR-301	Human IgG1	*S. aureus* alpha-hemolysin	II	NCT03816956
	ASN100	Mix of 2 human IgG1	Bi-component toxins & alpha-hemolysin	II	NCT02940626
	Tefibazumab	Humanized IgG1	S. aureus ClfA	II	NCT00198289
	MEDI4893	Human IgG1 modified	*S. aureus* alpha-hemolysin	II	NCT02296320
	514G3	Human IgG3	*S. aureus* protein A	II	NCT02357966
	Pagibaximab	Chimeric IgG1	Lipoteichoic acid	II	NCT00646399
	Aurograb	scFv	GrfA (lipoprotein)	III	NCT00217841
Multiple species	F598	Human IgG1	Poly-N-acetylglucosamine	II	NCT03222401

* STEC, Shiga toxin-producing Escherichia coli.

## The beginning of monoclonal antibody discovery and development

**Figure 1:**
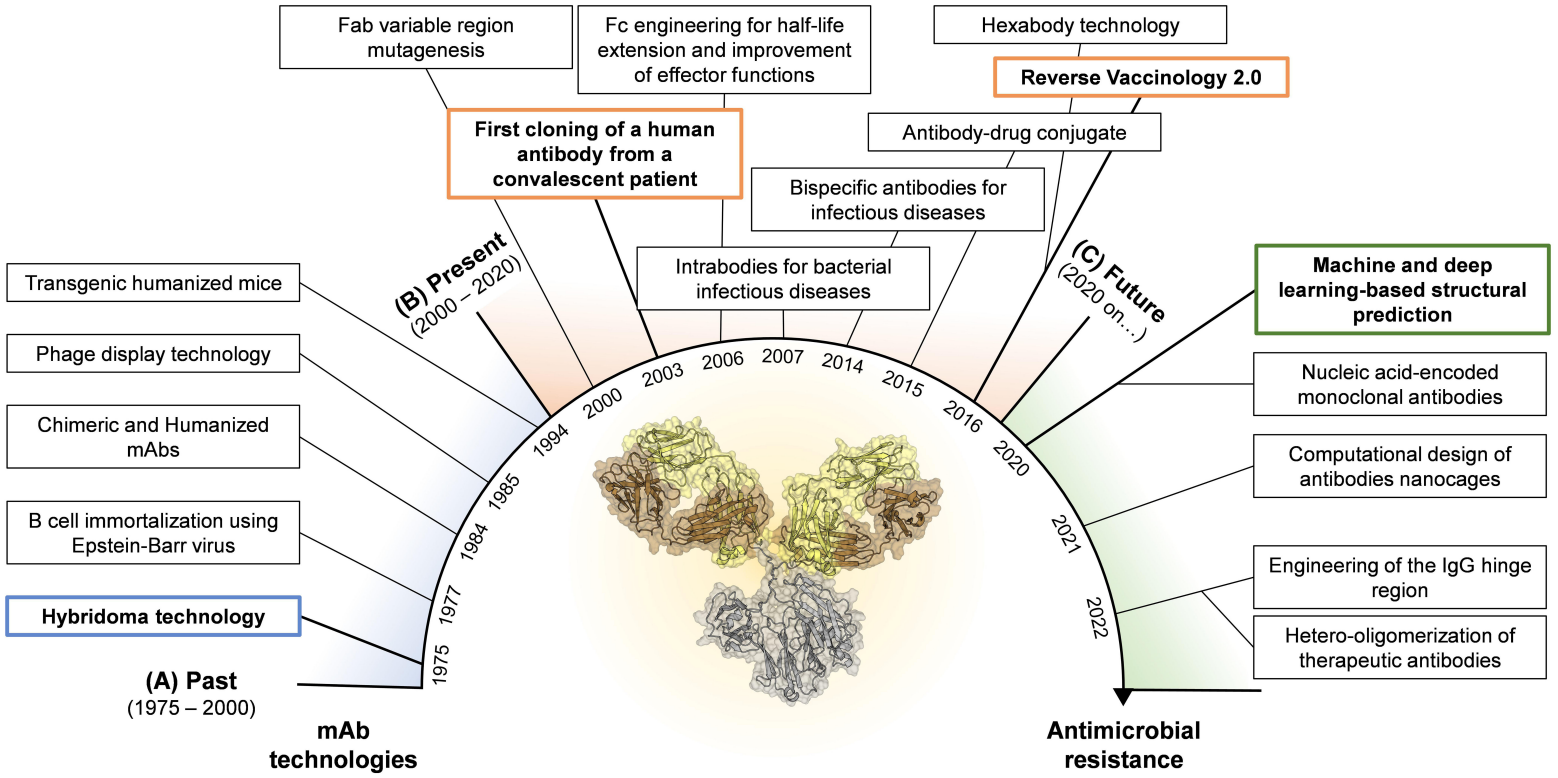
Timeline for mAb technologies developed in the past **(A)** and presently used in the field of infectious diseases **(B)**. Highlight of novel technologies that show great potential and could be applied in the near future in the fight against AMR **(C)**. Boxes highlighted in blue, orange and green represent the milestones achieved in the past, present, and future, respectively.

The journey for mAb discovery and development started in 1975 when Kohler and Milstein introduced the hybridoma technology (Köhler and Milstein, 1975) (Figure 1A). This approach consisted in the fusion of myeloma cell lines with splenocytes isolated from mice previously exposed to the antigen of interest leading to the formation of murine hybridomas able to produce antigen-specific antibodies. Despite this methodology led to the food and drug administration (FDA) approval of the first therapeutic mAb in 1986 (OKT-3, used to prevent organ transplant rejection) (Brekke and Sandlie, 2003), the fully murine origin of these antibodies was problematic as they did not recognize the human complement and Fc receptors, and elicited human anti-mouse antibodies leading to fatal antibody responses following their administration (Clark, 2000; Bruhns, 2012). Almost in concomitance with the hybridoma technology, Steinitz and colleagues developed another technique to produce human mAbs. In 1977 the authors described a novel approach based on the ability of the Epstein Barr virus (EBV) to convert normal human B lymphocytes and producing immortalized antibody-producing cell lines (Steinitz et al., 1977; Corti and Lanzavecchia, 2014). This approach opened for the first time the possibility to isolate human mAbs maintaining the characteristics of the original human B cells overcoming the limits of the hybridoma technology. EBV-immortalization showed its full potential in the field of infectious diseases almost 30 years later, when in 2003, Traggiai and coworkers identified the first human mAb against the severe acute respiratory syndrome coronavirus (SARS-CoV) (Traggiai et al., 2004). The limitation of this approach was the suboptimal immortalization of B cells, which plateaus at approximately 35% (Traggiai et al., 2004). To overcome the intrinsic immunogenicity of murine mAbs and increase the efficiency of antibody discovery, two different technologies were developed. In 1984, the hybridoma technique was combined with genetic engineering to generate partial human mAbs. Initially, chimeric mAbs were generated by substituting the mouse with the human constant region (Fragment crystallizable region; Fc) (Boulianne et al., 1984; Morrison et al., 1984). Following, using the same approach, Jones and colleagues replaced also portions of the murine variable region of chimeric mAbs to generate fully humanized antibodies (Jones et al., 1986). Although the human homology was 75% and 95% for chimeric and humanized mAbs respectively, these classes of antibodies were not able to completely avoid anti-antibody responses raised after injection (Jones et al., 1986; Clark, 2000). The breakthrough of phage display technology, introduced by Smith et al. in 1985, allowed to circumvent the immunogenicity issue leading to the production of fully human mAbs. This technology was based on the construction of phage display libraries based on human immunoglobulin G (IgG) heavy and light chain variable region sequences derived from immunized and infected individuals, or by synthetic libraries (McCafferty et al., 1990). The human variable regions were introduced in the Gene III of filamentous bacteriophages that express the antibody on their surfaces and can be isolated by affinity chromatography using the antigen of interest as fishing bait (Smith, 1985; McCafferty et al., 1990). In 1991, this approach led for the first time the identification of 15 different mAb clones specific for the glycoprotein 120 (gp120) protein of the human immunodeficiency virus (HIV), opening the road for an antibody based therapeutic approach against retroviruses (Burton et al., 1991). Anyway, the phage display library approach had two major downsides. Firstly, the production of mAbs did not represent the natural antibody repertoire since mAbs were generated by random pairing of the heavy and light chains (Novobrantseva, 2005). Secondly, the isolation of these mAbs was based on prior knowledge of targeted antigens. In 1994, Lonberg and colleagues introduced another method to isolate and produce fully human mAbs. The authors developed transgenic humanized mice, called “HumAb” mice, by using a genetic homologous recombination approach to disrupt the mouse immunoglobulin genes and replace them with components of human heavy and light chain transgenes, which included the constant and variable (V), diversity (D) and joining (J) regions (Scott, 2007). Produced humanized mice were then immunized against the antigen of interest and antigen-specific B cells were isolated and mAbs produced by the traditional hybridoma production systems.

All the approaches implemented in over two decades of technological advancement, led to the development of only five therapeutic mAbs against infectious agents. In 1998, the FDA approved the first humanized mAb (palivizumab) that targeted the RSV fusion (F) protein (Maeurer et al., 2016). Much later, the FDA approved the phage display-derived mAb raxibacumab (2102) and the humanized antibody obiltoxaximab (2016) to treat inhalation of the *Bacillus anthracis* exotoxin responsible for the lethal anthrax disease (Table 1) (Tsai and Morris, 2015; Reichert, 2016). In the same year, the mAb preparation Rabishield, and later in 2019 the mouse-derived mAb cocktail Twinrab, were marketed in India to treat rabies virus infection (Fan et al., 2022). These antibodies, while highlighting the promise of mAbs for therapy against infectious diseases, showed the limits of these initial technologies as all mAbs displayed a very low functional activity resulting into high dosage, need of long intravenous administration and scarce accessibility especially in low-middle income countries.

## The modern era of monoclonal antibody technologies

The limits posed by the initial technologies highlighted the need to develop novel approaches to ameliorate the functional properties of mAbs already discovered as well as to identify extremely potent human mAbs as therapeutics (Figure 1B). The explosion of molecular biology techniques and structural based approaches in the late 1990's—early 2000's enabled an unprecedented possibility to manipulate antibody molecules which paved the way to the engineering of the fragment antigen binding (Fab) and fragment crystallizable (Fc) regions (Liu et al., 2020; van der Horst et al., 2020). Fab engineering allowed to increase the antibody affinity to its cognate antigen. Two main approaches were developed to achieve this goal and were the display- or structure-based methods. The display-based method relies on building libraries of variants by using an error-prone PCR which introduces random mutations in the antibody Fab region. These mutations can be inserted randomly in the whole variable region, as successfully described in 2002 by Maynard and colleagues which used this strategy to generate a panel of toxin-neutralizing antibodies against *Bacillus anthracis* (Maynard et al., 2002), or to selectively mutate the antibody complementary determining regions (CDRs) of the antibody, as reported by Yang and coworkers which exploited this approach to increase the binding affinity of a potent human anti-HIV-1 monoclonal antibody (Yang et al., 1995). Differently, the structure-based method for Fab engineering and affinity enhancement relies on the analyses of complex antibody-antigen structures and the modification of specific contact sites in the antibody variable region. The structure-based method has been widely used in the field of mAbs against viral pathogens like HIV, influenza, and dengue virus (Zhou et al., 2010; Lingwood et al., 2012; Robinson et al., 2015; Sarker et al., 2022). While the purpose of Fab modifications was to increase the mAb ability to bind its cognate antigen, Fc engineering aimed to enhance the antibody pharmacokinetics and effector functions. Indeed, a crucial aspect for therapeutic mAbs is the serum half-life which needs to be extended beyond the ~23 days of natural IgGs (Saxena and Wu, 2016). In 2006, Dall'Acqua and colleagues demonstrated that serum half-life, tissue distribution and activity of a given human IgG1 can be modulated by the introduction in the Fc region of the YTE (M252Y/S254T/T256E) mutations (Dall'Acqua et al., 2006). These mutations were later used to modify an anti-RSV mAb, named motavizumab (MEDI8897), tested in 2015 in a phase 1b/2a clinical trial, which resulted in extended half-life of up to 150 days in healthy preterm infants (Domachowske et al., 2018). Fc region has also been the target for improved effector functions. It is worth to mention that four different set of mutations developed between 2006 and 2012 known as LS (M428L/N434S) (Zalevsky et al., 2010), AAA (T307A/E380A/N434A) (Petkova et al., 2006), QL (T250Q/M428L) (Hinton et al., 2004) and V308P (Datta-Mannan et al., 2012), which improve antibody half-life and antibody-dependent cellular cytotoxicity (ADCC) and complement-dependent cytotoxicity (CDC) activity (Booth et al., 2018). Furthermore, by delivering Fc-Fc gamma receptor (FcγR) and Fc-C1q interactions, mAbs can link exquisite specificity to power cellular and complement-mediated effector functions. This biological function was exploited by De Jong and colleagues in 2016, which identified, among others, the mutation E430G that enhances hexamer formation and complement activation by IgG1 antibodies (de Jong et al., 2016). This technology was applied in the context of bacterial infections, by Chakraborti and colleagues to test the efficacy *in vitro* and *in vivo* of a human IgG1 chimeric mAb, named 2C7, against *Neisseria gonorrhoeae* (Chakraborti et al., 2020). Another engineering approach that was introduced to increase the antibody efficacy and breadth of protection was the production of IgG mixture and bispecific antibodies (bsAbs) (Nie et al., 2020). Despite the possibility to develop bsAbs emerged in 1998 thanks to the knobs-into-holes approach (Merchant et al., 1998), it wasn't until 2014 that DiGiandomenico and colleagues developed a bispecific mAb against *Pseudomonas aeruginosa*, BiS4αPa, by merging in one molecule an anti PcrV mAb and an anti Psl mAb. PrcV is a type III secretion, toxin injectosome while Psl is a serotype independent exopolysaccharide crucial for colonization and biofilm formation (DiGiandomenico et al., 2014). In 2015, antibody–antibiotic conjugate (AAC) was also introduced as potential approach to treat bacterial infections. This method combines the key attributes of both antibody and antibiotic in a single molecule (Mariathasan and Tan, 2017). A specific AAC has been recently reported to show promise in the treatment of *Staphylococcus aureus* which showed to be superior to vancomycin for treatment of bacteremia (Lehar et al., 2015).

In addition, scientists have developed several approaches to generate antibodies able to reach and withstand the intracellular environment (Slastnikova et al., 2018). Since the 1990s, intrabodies have been introduced in different research areas to inhibit the activity of selected intracellular antigens (Biocca and Cattaneo, 1995; Cattaneo and Biocca, 1999). The technology has been later ameliorated and applied to prevent bacterial conjugation and cell damage by bacterial toxins (Carcillán-Barcia et al., 2007; Tremblay et al., 2010; Alzogaray et al., 2011; Li et al., 2015). Indeed, intrabody expression in Vero cells has been shown to block *Clostridium difficile* TcdB-mediated cell intoxication (Li et al., 2015). It is worth noting that AMR spreads through horizontal gene transfer (HGT) and it is associated with intracellular bacterial antigens, which could be potentially targeted by intrabodies-mediated applications (Slastnikova et al., 2018; Lerminiaux and Cameron, 2019). All the approaches above described where used to improve mAbs discovered through the use of approaches developed in the 1970's−1990's, but one last step forward allowed to change the game of mAb discovery. Indeed, the enormous advancement in human B cell sequencing, single cell isolation, high-throughput screening and structural characterization of protective antigens has provided the molecular and mechanistic understanding which led to the Reverse Vaccinology 2.0 approach in 2016 (Rappuoli et al., 2016). This approach relies on the identification of extremely potent mAbs directly from the blood of convalescent or immunized human donors, which showed a neutralization activity over 10,000-fold higher compared to those initially discovered through phage display or hybridoma technologies (Sok and Burton, 2018). The reverse vaccinology 2.0 approach is now widely used in the field of infectious diseases, and it was exploited to generate therapeutic mAbs against a variety of viral pathogens including HIV, influenza, RSV, HCMV, Ebola, dengue virus, zika virus, and more recently SARS-CoV-2 (Beltramello et al., 2010; Corti et al., 2010; Macagno et al., 2010; Krause et al., 2011; de Alwis et al., 2012; Pinto et al., 2020; Zost et al., 2020; Andreano et al., 2021). This approach is currently being used to discover therapeutic mAbs against AMR-bacteria.

## The future of therapeutic monoclonal antibodies against antimicrobial resistant bacteria

The technologies described in the previous paragraph enabled the discovery of potent mAbs against infectious diseases especially in the field of viral pathogens. However, with the main aim of increasing the efficacy of therapeutic mAbs against bacterial targets, many efforts were recently made. Progresses in the field include machine and deep learning approaches for structural prediction (Jumper et al., 2021), the design computational design of antibody nanocages (Divine et al., 2021), the implementation of mAb immunomodulatory activities through the engineering of the IgG hinge region (Foss et al., 2022), the development of nucleic acid-encoded monoclonal antibodies (Schlake et al., 2019) and the hetero-oligomerization of therapeutic antibodies (Oostindie et al., 2022) (Figure 1C). Herein, we discussed novel approaches that show great potential in the fight against AMR.

**Machine and deep learning for protein structure prediction:** At the end of 2020, the DeepMind's program called AlphaFold2 showed the outstanding ability to predict protein structures starting from their amino-acid sequences with startling accuracy (Callaway, 2020). While AlphaFold2 outperformed its competition in 2020, it was only in July 2021 that the manuscript describing the method and source code was released (Jumper et al., 2021). Of course, its potential to identify therapeutic mAbs, the ability to predict antibody-antigen interactions and the possibility to computationally design immunoglobulin-like molecules were immediately grasped. In fact, Robinson and coworkers already at the end of 2021, quickly exploited this method to computationally identify mAbs that rearranged in similar structures and engaged the same neutralizing epitope on the SARS-CoV-2 spike protein despite having different amino acidic sequences. The potential of this approach goes far beyond the mere structural prediction of mAbs, in fact the possibility to computationally define protein-protein interaction was by far one of the most desired technologies in the field of mAb and vaccine development. Machine and deep learning approaches could be exploited to predict interactions between mAbs and the bacterial proteome of surface exposed targets to identify antigens that could elicit a protective antibody response and function as optimal vaccine candidates. Recently, approaches like AlphaFold-multimer (March 2021), Absolut! (July 2022) and AbAdapt (September 2022), aimed at improving the prediction of antibody-antigen interactions to make machine and deep learning approaches suitable to achieve this goal (Evans et al., 2022; Robert et al., 2022; Xu et al., 2022). Finally, computational models for high-resolution structural validation have also been recently used for *de novo* design of antibody-like domains. The approach developed in October 2022 by Chidyausiku and coworkers aimed to overcome the limits of mAb engineering by designing antibody-like scaffolds, not produced in nature, to graft loops containing the CDRs of functional antibodies (Chidyausiku et al., 2022). The gargantuan leap in solving three-dimensional structure of proteins marked a pivotal milestone in the field of mAb discovery, design and development which could be instrumental in the fight against AMR.

**Antibodies cages (AbCs):** Multimerized antibodies that include several antigen binding sites are a potent tool of considerable interest in the field of therapeutic mAbs. In a recent study, Divine and colleagues established a new method, applicable to IgG antibodies, to increase antibodies valency through antibodies protein nanoparticles (Divine et al., 2021). The technique is based on the computational design of proteins that direct the assembly of selected antibodies in well-defined nanocages which can recognize a larger target compared to a single antibody. The antibody nanocages (AbCs) are composed of a Fc fusion or an antibody and a homo-oligomer that controls Fc-binding and nanocages assembly. AbCs have different structures from dihedral to icosahedral architectures and therefore include from 2 to 30 antibodies per nanocage. Divine and colleagues elegantly demonstrated that AbCs are superior to free antibodies in several immune responses including activation of tumor cell apoptosis and angiogenesis, B cell activation, and neutralization of SARS-CoV-2 pseudovirus. AbCs offer interesting advantages compared to previous antibody mixture approaches because of their controlled formulation and increased homogeneity which will likely have an impact on their use as a therapeutic tool.

**HINGEneering:** Recent studies have demonstrated that the molecular engineering of the hinge region is a valuable tool to modulate the functionality of IgG antibodies (Foss et al., 2022; Orr et al., 2022). IgG3 is a very potent immunoglobulin, which can trigger different effector functions, including complement activation, antibody-mediated phagocytosis and antibody-mediated cellular toxicity (Damelang et al., 2019). In their study, Foss and colleagues have demonstrated that the more extended IgG3 hinge region associates with enhanced intracellular antiviral immunity compared to the other IgG subclasses. This process occurs *via* the tripartite motif containing 21 (Trim21) Fc receptor, an intracellular detection system for opsonized pathogens that have evaded lysosomal degradation (Foss et al., 2022). Although Trim21 binds specifically to the Heavy chain constant domain CH2-CH3 and not to the mAb hinge region, a substitution of the IgG3 hinge through molecular engineering to the other IgG subclasses boosted the Trim21 activation, leading to pathogen clearance. Moreover, the IgG3 hinge-engineering onto the IgG1 subclass increases complement deposition upon pathogen opsonization (Foss et al., 2022). To date, IgG3 has not been the choice of interest for marketed mAbs because of concerns about its short half-life and increased immunogenicity, however, substituting the IgG3 hinge region into the IgG1 structure may overcome these limitations and it is a promising approach to developing future antibody therapeutics (Chu et al., 2021; Werner and Nimmerjahn, 2022).

**Nucleic acid-encoded monoclonal antibodies:** The possibility of expressing proteins from nucleic acids *in vivo* was first described in the 1990 by Wolff et al. (1990). The first preclinical evaluation of an mRNA-encoded vaccine instead dates to 1993, when Martinon and colleagues developed and tested in mice an experimental vaccine against influenza consisting in a liposome-encapsulated mRNA encoding the influenza virus nucleoprotein sequence (Martinon et al., 1993). The discovery of innovative vehicles allowing RNA or DNA delivery to human cells *in vivo* plus the optimization of RNA synthesis purification platforms (Karikó et al., 2005, 2011), led to a breakthrough in 2020, when two mRNA-based vaccines against SARS-CoV-2 were the first-in-class approved products available worldwide to fight the ongoing COVID-19 pandemic (Polack et al., 2020; Baden et al., 2021). The rapid launch of these mRNA-based COVID-19 vaccines provided the first evidence of the advantages of nucleic acid-based therapeutics compared to classical, protein-based products (Jackson et al., 2020). The combination of nucleic acid technologies with the field of monoclonal antibodies in the field of infectious diseases was only recently explored. Notably, August and colleagues published in 2021 the first results of a Phase I clinical trial in human, in which an anti-Chikungunya mRNA-encoded mAb has been administered intravenously to patients, to evaluate safety and pharmacokinetics in healthy adults (August et al., 2021). In the field of antibacterial mAbs, one of the first notable examples of nucleic acid-encoded mAb was provided by Parzych and colleagues, who developed a DNA version of their chimeric mAb 2C7 against *Neisseria gonorrhoeae* which resulted in protection from infection until 65 days post-administration (Parzych et al., 2021). All these described examples provided encouraging preliminary evidence supporting further research in the field of nucleic acid-encoded mAbs.

**HexElect**: Single antigens targeted by therapeutic mAbs may not provide sufficient selectivity to distinguish between commensal and pathogenic bacteria. The possibility to develop an approach that enhances the functional selectivity of therapeutic antibodies by making their activity dependent on clustering after binding to two different antigens is extremely appealing for mAbs against bacterial pathogens. In a recent study Oostindie and coworkers developed a new method named HexElect which suppresses individual homo-oligomerization of two distinct mAbs (IgG1-Campath-RGE and IgG1- IgG1-11B8-AGK) while promoting their pairwise hetero-oligomerization after binding co-expressed antigens on the surface of the targeted cells (Oostindie et al., 2022). Upon hetero-oligomerization, the complex triggers complement or cell-mediated effector functions to kill the target. The authors were able to reach hetero-complexes formation and maximize C1q affinity and recruitment by introducing in the Fc region of selected antibodies two specific point mutations in addition to E430G, named K439E or S440K for hetero-complexes, and G236R or G237A for complement activation. Despite this approach was used to study the effect of therapeutic mAbs against B cell tumors, the HexElect approach results to be a new and promising strategies also in the field of infectious diseases.

## Conclusions

The technological and methodological advancements of the last 50 years brought the field of mAb discovery and engineering at their peaks. Despite that, only 26 mAbs against bacterial pathogens have so far reached clinical phases for efficacy evaluation and only five were approved by the FDA. Therefore, a further step forward needs to be made. The new technologies herein described can contribute to this effort and finally allow monoclonal antibodies to find their place in the fight against AMR.

## Author contributions

All authors contributed to the conceptualization, writing, and final revision of this manuscript.
